# The Income Tax to Be Paid by Physicians

**Published:** 1863-11

**Authors:** 


### THE INCOME TAX TO BE PAID BY PHYSICIANS.

The Committee appointed at the last meeting of the Medical Association of the District of Columbia have obtained the information with regard to the Income Tax, embodied in the following letter:

(Official.)

TREASURY DEPARTMENT, OFFICE OF INTERNAL REVENUE,
WASHINGTON, June 11th, 1863.

GENTLEMEN:—Your letter of this date has been received, and contents noted. It is asked whether an assessment for Income Tax is to be made upon collections during the year 1862, for professional services rendered during that year and previous years, and whether an estimate of unrealized, or contingent income due for services rendered in that year, ought to be included? I answer, that the assessment should be made upon all collections during the year 1862, without regard to whether the services were rendered during that or previous years. If any profits made during that year and uncollected, remain uncollected when they might have been readily realized, and with a view merely to avoid the assessment of the tax, they are to be considered as collected, and assessed accordingly; for no evasion of the liability of the tax-payer of his duty under the law, should be allowed to profit him. But merely contingent profits, uncollected, the sum not ascertained, remaining open for adjustment, are not liable to assessment.

2d. As to "expenses necessarily incurred in carrying on any trade, business, or profession," physicians cannot be allowed the wear and tear of horses, carriages and harness, any more than they can of their own constitutions, or of their health, necessarily injured in the practice of their vocation; but any incidental expenses, such as the feeding of horses, hire of servants, and such like, are to be deducted from this income.

Very respectfully,

JOSEPH J. LEWIS, *Commissioner.*

